# 68 and FX2149 Attenuate Mutant LRRK2-R1441C-Induced Neural Transport Impairment

**DOI:** 10.3389/fnagi.2016.00337

**Published:** 2017-01-10

**Authors:** Joseph M. Thomas, Tianxia Li, Wei Yang, Fengtian Xue, Paul S. Fishman, Wanli W. Smith

**Affiliations:** ^1^Department of Pharmaceutical Sciences, University of Maryland School of PharmacyBaltimore, MD, USA; ^2^Department of Neurology, University of Maryland School of MedicineBaltimore, MD, USA; ^3^Neurology Service, VA Maryland Healthcare SystemBaltimore, MD, USA; ^4^Department of Psychiatry, Johns Hopkins University School of MedicineBaltimore, MD, USA

**Keywords:** LRRK2, Parkinson's disease, neural transport, GTP binding inhibitor, mitochondria, lysosomes

## Abstract

Leucine-rich repeat kinase 2 is a large protein with implications in genetic and sporadic causes of Parkinson's disease. The physiological functions of LRRK2 are largely unknown. In this report, we investigated whether LRRK2 alters neural transport using live-cell imaging techniques and human neuroblastoma SH-SY5Y cells. Our results demonstrated that expression of the PD-linked mutant, LRRK2-R1441C, induced mitochondrial, and lysosomal transport defects in neurites of SH-SY5Y cells. Most importantly, recently identified GTP-binding inhibitors, 68 and FX2149, can reduce LRRK2 GTP-binding activity and attenuates R1441C-induced mitochondrial and lysosomal transport impairments. These results provide direct evidence and an early mechanism for neurite injury underlying LRRK2-induced neurodegeneration. This is the first report to show that LRRK2 GTP-binding activity plays a critical role during neurite transport, suggesting inhibition of LRRK2 GTP-binding could be a potential novel strategy for PD intervention.

## Introduction

Parkinson's disease (PD) is a common progressive neurodegenerative disease with movement disorder. Human PD cases often display a loss of dopaminergic neurons in the substantia nigra and the presence of protein inclusions (Lewy bodies) in the brain (Dauer and Przedborski, 2003; Cookson, 2010). Although the pathogenesis of PD is not completely clear, mutations in the leucine-rich repeat kinase 2 (LRRK2) represent the most common known cause of PD to date (Klein and Lohmann-Hedrich, 2007; Paisán-Ruiz et al., 2013). LRRK2-associated PD cases have pleomorphic pathology, however the physiological function of LRRK2 is largely undetermined. LRRK2 is a large 286 kDa cytoplasmic protein and contains several functional domains (Li et al., 2011). Studies suggest that LRRK2 is involved in multiple cellular processes, including cytoskeletal organization, neuronal outgrowth, mitochondrial dynamics, autophagy, endocytosis, and protein interactions (Mata et al., 2006; Raquel Esteves et al., 2014). PD-linked LRRK2 mutations cause neuronal degeneration although the underlying mechanisms remain elusive.

Disease-linked mutations mostly occur in one of LRRK2's two enzymatic sites: a GTPase domain and a kinase domain (Lee et al., 2012; Martin et al., 2014). These regions have shown considerable overlap and interplay in PD pathology in previous studies (Gilsbach and Kortholt, 2014; Raquel Esteves et al., 2014). The majority of LRRK2 PD mutations can lead to increased kinase activity and altered GTP binding/GTPase activities (Smith et al., 2006; Xiong et al., 2012). The LRRK2-R1441C mutation occurs in the GTPase domain, and mutations at this residue have previously shown a combination of decreased GTPase activity, a possible increase in GTP-binding affinity, and a subsequently overactivated kinase activity (Xiong et al., 2012; Liao et al., 2014; Raquel Esteves et al., 2014). Studies on LRRK2 kinase and GTPase domain inhibitors suggest that LRRK2 kinase and GTPase functions play critical roles in neurodegeneration. Our recently identified novel GTP-binding inhibitors (Li et al., 2014, 2015) provide useful pharmacological probes to investigate LRRK2 pathophysiological roles in PD pathogenesis.

Neural transport, in particular axonal transport, is especially necessary in maintaining healthy neurons as neurons are highly polarized cells which utilize specific motor proteins to travel in directions away from the cell body (anterograde) or toward the cell body (retrograde) along cytoskeletons composed of microtubules (Fu and Holzbaur, 2014; Maday et al., 2014). Defects in axonal transport can lead to disruption in energy homeostasis, impaired protein clearance pathways, neurite shortening, and eventual cell death (Fu and Holzbaur, 2014; Maday et al., 2014). Impaired neural transport has been shown to contribute to the pathogenesis of neurodegenerative disorders, including Alzheimer's disease (AD), amyotrophic lateral sclerosis (ALS), and hereditary spastic paraplegia (HSP) (Chevalier-Larsen and Holzbaur, 2006). Recent studies suggest that neural transport (especially axonal transport) impairment may also contribute to mutant alpha-synuclein toxicity and have implications in PD pathology (Kim-Han et al., 2011; Lu et al., 2014; Koch et al., 2015). LRRK2 has been previously shown to interact with microtubules and some cytoskeleton proteins (Kawakami et al., 2012; Caesar et al., 2013; Beilina et al., 2014; Law et al., 2014). A recent study has reported that LRRK2 interacts with a group of Rab GTPases that play critical roles in cell membrane trafficking (Dodson et al., 2012; Steger et al., 2016). Computer based predictions of LRRK2's interactome suggest that a cluster of its interactors are more likely involved in regulating cell transport/localization and cell organization (Manzoni et al., 2015). LRRK2 has been shown to regulate neuronal outgrowth and intracellular events necessary for maintaining healthy neurons (Ramonet et al., 2011; Su and Qi, 2013).

In this study, we are interested in investigating whether PD-linked LRRK2 mutations alter neural transport and whether inhibition of LRRK2 enzymatic activities plays a role in this process. We observed the movement of transport cargos including mitochondria and lysosomes in differentiated neuroblastoma SH-SY5Y cells by using live-cell-imaging approaches. Our studies not only provide the mechanisms of early neurodegeneration but also provide a potential novel therapeutic strategy for intervention.

## Materials and methods

### Materials and reagents

Cell culture media and transfection reagents (LipofectAMINE and Plus reagent) were purchased from Invitrogen (Carlsbad, CA). Compound 68 was custom ordered from Chembridge and FX2149 and FX2151 were synthesized by Dr. Fengtian Xue's group. Compounds were dissolved in 0.1% DMSO for live-cell imaging and GTP-binding experiments. MitoTracker Orange and LysoTracker Red were from Molecular Probes at Thermo Fisher Scientific, and were dissolved in DMSO for live-cell imaging experiments.

### Cell culture and LRRK2 transfection

SH-SY5Y human neuroblastoma cells (passage 4–10) were from ATCC (Manassas, VA, USA), contain dopamine, and were grown in OptiMem I media with 10% FBS and antibiotics as described previously. In this condition, SH-SY5Y cells display differentiated neuron features with neuritic processes for neural transport studies (Plowey et al., 2008). The Flag-wild-type and Flag-R1441C pcDNA3.1-LRRK2 constructs were described previously (Smith et al., 2006; Ko et al., 2009). pMax-GFP was from Clontech. Transfections were performed using Lipofectamine™ and PLUS™ Reagents (Invitrogen) according to the manufacturer's recommendations.

### LRRK2 neuritic injury assays

SH-SY5Y cell neuritic injury assays were conducted as described (Smith et al., 2005; Yang et al., 2012). GFP and various pcDNA3.1-LRRK2 plasmids were transfected into SH-SY5Y cells at a 1:15 ratio for 24 h in OptiMem I media supplemented with 10% FBS and then changed to DMEM with N2 supplement for an additional 24 h. Under these conditions, above 85% of GFP positive neurons also expressed LRRK2 variants by anti-LRRK2 immunostaining as described previously (Smith et al., 2005). Fluorescence microscopy was used to measure cell viability fields by counting the healthy viable cells, defined by possessing at least one smooth extension (neurite) twice the length of the cell body from nine randomly selected fields. The experiments were repeated three times in duplicate. The quantification for LRRK2 neurite injury was performed by an investigator blinded to transfection groups.

### GTP binding activity assay and western blot analysis

SH-SY5Y cells transiently transfected to express wild-type, LRRK2-R1441C, or empty vector as described above were harvested. Lysates were incubated with 68 (100 nM) for 1 h at 4°C, then precipitated with GTP-agarose (Sigma) for an additional 1 h at 4°C. Agarose samples were diluted in 2X Loading buffer (Invitrogen) and 3% βME and denatured in 85°C for 5 min. Samples were resolved using 4–12% NuPAGE Bis-Tris gels and subjected to Western blot analysis using anti-Flag and anti-actin antibodies (Sigma) (Li et al., 2014, 2015).

### Live-cell imaging and transport analysis

Procedures were performed as described previously with slight modification (Cherra et al., 2013; Das et al., 2014; Klinman and Holzbaur, 2015). SH-SY5Y cells were plated and cultured for 1 day prior to transfection. Cells were co-transfected with various LRRK2 plasmids and pMax-GFP at a 1:15 ratio using Lipofectamine™ and PLUS™ Reagents (Invitrogen) according to the manufacturer's protocol. After 4 h of transfection, the cells were maintained in 10% FBS OptiMem I medium for 48 h, then fluorescent dye [MitoTracker Orange (125 nM) or LysoTracker Red (100 nM)] was added for 30 min. Before imaging, the cells were washed once with phenol red-free OptiMem I and kept in this phenol red-free media for transport assays. For inhibitor treatment, 68 (100 nM), FX2149 (100 nM), FX2151 (100 nM), or 0.1% DMSO was added to growth media 4 h post-transfection, and replaced every 12 h for a total of 48 h prior to imaging. Images were taken using a Zeiss Avixon Camera under a computer controlled fluorescent microscope. Images were taken every 15 s for a total of 5 min. Images were compiled into a time-lapse sequence using NIH ImageJ software, and fluorescent puncta in healthy cell neurites were tracked using the MTrackJ plugin (Biomedical Imaging Group Rotterdam, Meijering et al., 2012). Puncta were classified as anterograde (≥2 micron displacement away from cell body), retrograde (≥2 micron displacement toward cell body), or stationary (<2 microns displacement). Puncta were recorded at their farthest displacement in either direction over the course of the time-lapse sequence, and puncta were counted as both anterograde and retrograde if a displacement of at least 2 microns occurred in the opposite direction during this time. To focus specifically on the interruption of highly processive motility, three puncta per cell traveling the farthest in both anterograde and retrograde directions were tracked for run lengths.

### Data analysis

Quantitative data represent arithmetic means ± SEM from at least three separate experiments for each condition. Live-cell images were taken for at least three separate experiments with at least four cells per experiment for each condition. Only viable GFP-expressing cells were included in these analyses. Statistically significant differences among groups were analyzed by ANOVA with post hoc Tukey's test using Sigmastart 3.1 statistical software. A *p* < 0.05 was considered significant.

## Results

### LRRK2-R1441C caused neurite injury in SH-SY5Y cells

**Figure 1:**
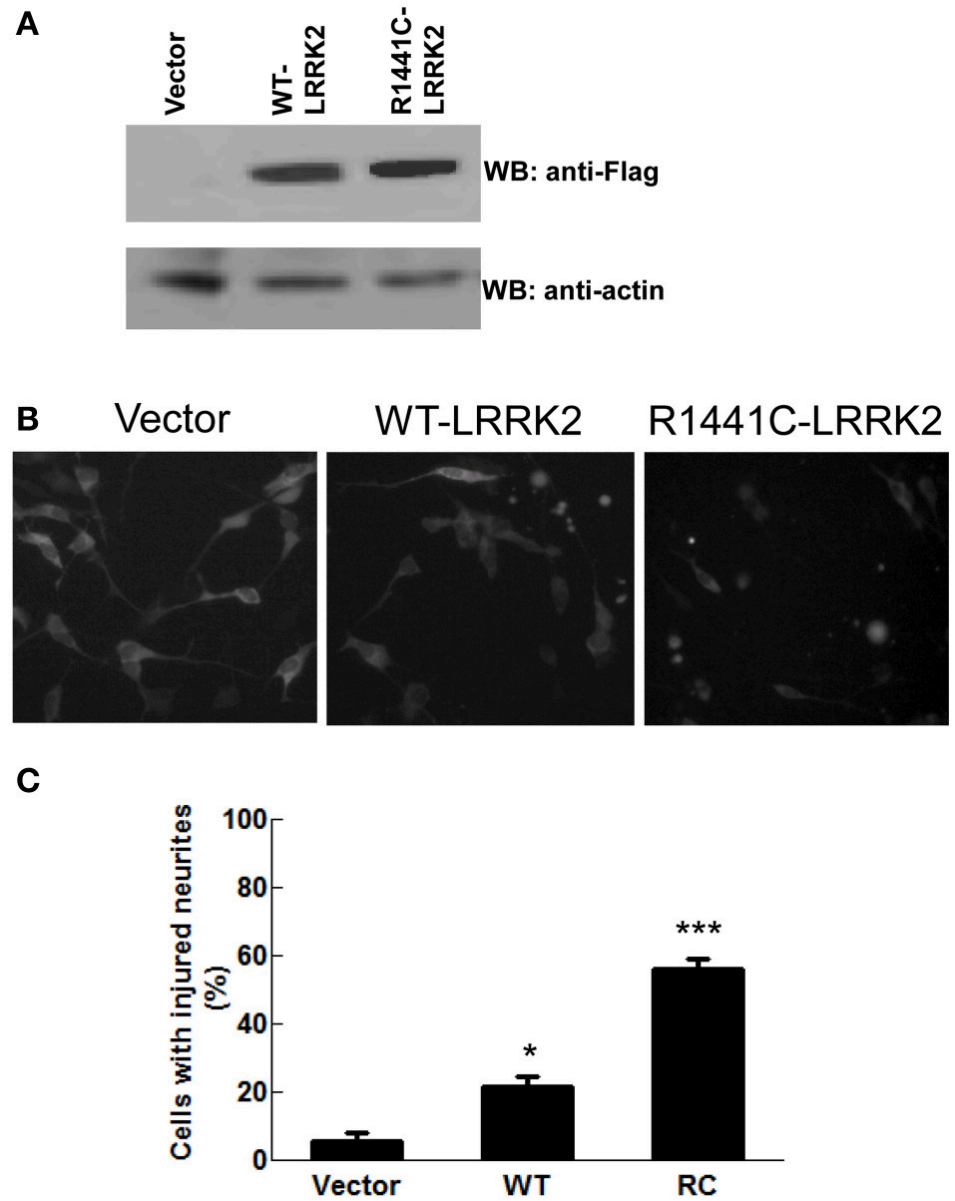
**LRRK2-R1441C mutation caused neurite injury in PD cell model**. SH-SY5Y cells were co-transfected with GFP and various Flag-tagged pcDNA3.1-LRRK2 plasmids at a 1:15 ratio as described in the method section. **(A)** Cell lysates were harvested and subjected to western blot analysis using anti-Flag and anti-actin antibodies. **(B,C)** Two days post-transfection, cells were imaged for GFP fluorescence. Nine images were taken in separate fields for each experiment. **(B)** Shown are representative cell images in each experiment group. **(C)** Neurite injury was measured by dividing the number of viable GFP positive cells with at least one smooth extension equal to twice the length of the cell body and at least one neuritic swelling by the total number of healthy GFP-positive cells with at least one neurite (*F* = 70.88). ^*^p < 0.05 by ANOVA compared to vector control. ^***^p < 0.001 by ANOVA compared to vector control.

Previous studies reported that the LRRK2-R1441 mutant causes disrupted neurite outgrowth, leads to decreased neuritic branching in PD models using cultured cell lines or primary neurons (MacLeod et al., 2006; Heo et al., 2010; Cherra et al., 2013; Tagliaferro et al., 2015). Here, we used SH-SY5Y cells transiently transfected with Flag-wild-type and Flag-R1441C-LRRK2 constructs as a cell model to express the LRRK2 variants. SH-SY5Y were grown in the media (10% FBS, OptiMem I media) to display mature neuron morphology with smooth neurite processes. Western blot analysis showed that LRRK2 variants were expressed well after 2–3 days transfection (Figure 1A). Consistent with previous reports (Smith et al., 2005, 2006; Tagliaferro et al., 2015), cells expressing LRRK2-R1441C displayed noticeable neuritic injury (beading) and impaired neurite complexity after 48 h as compared with vector controls (Figure 1B). The percentage of cells with at least one neuritic injury (beading) was significantly increased in those expressing LRRK2-R1441C compared with empty vector control cells. Wild-type LRRK2 overexpression only displayed a moderate neuritic injury (Figure 1C).

### LRRK2-R1441C disrupted mitochondrial transport in SH-SY5Y neurites

**Figure 2:**
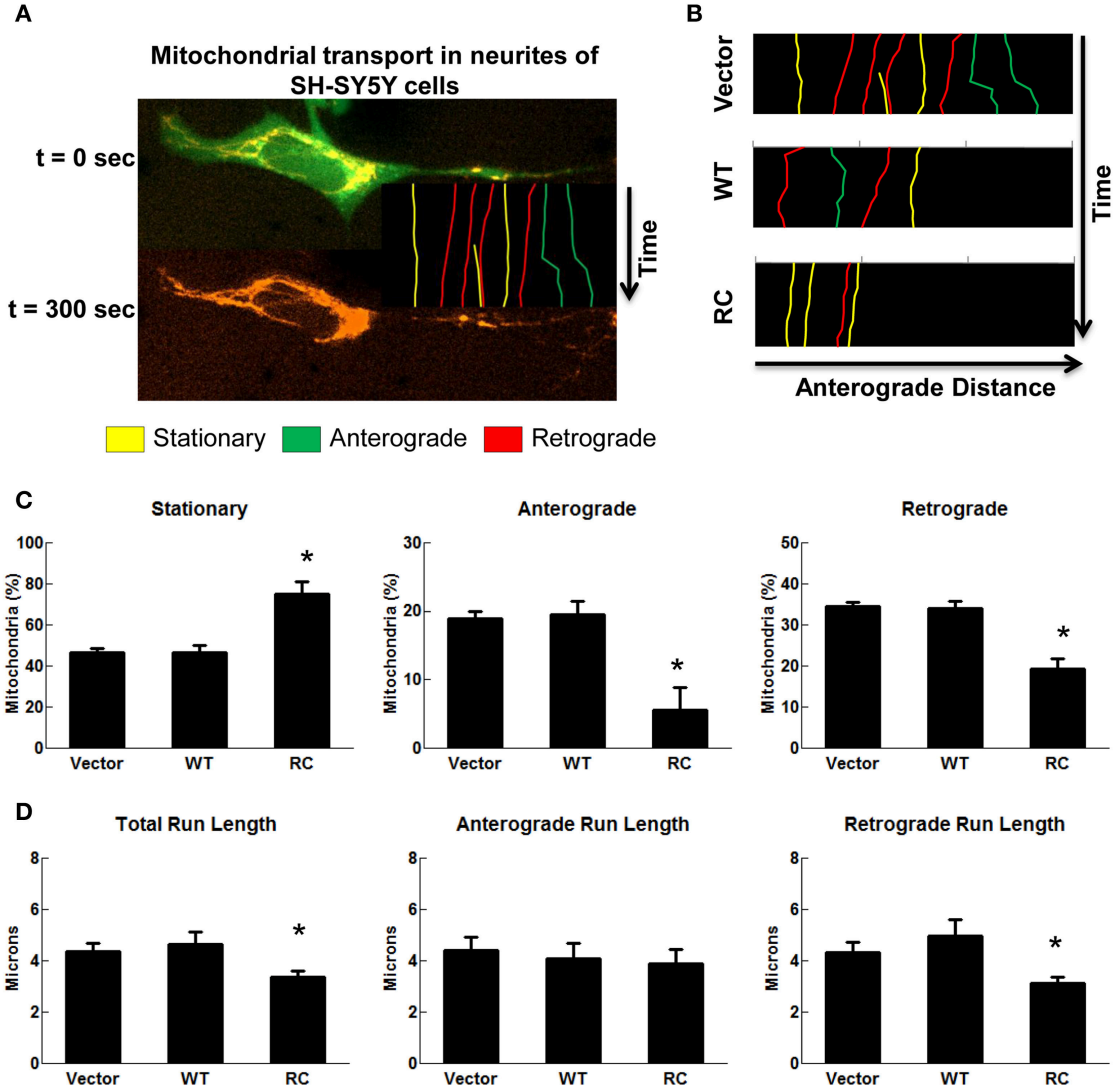
**LRRK2-R1441C induced neuritic mitochondria transport impairments**. SH-SY5Y cells co-transfected with GFP and various pcDNA3.1-LRRK2 plasmids at a 1:15 ratio for 48 h. Prior to imaging, cells were stained with MitoTracker Orange for 30 min. Cells were imaged every 15 s for a total of 5 min as mentioned in detail in methods section. **(A)** An image depicts time-lapse live-cell imaging technique used for measuring neuritic mitochondrial transport. Mitochondria in neurites of GFP-positive SH-SY5Y cells were traced using images captured during time progression, and classified as stationary (yellow), anterograde (green), or retrograde (red) as described in methods section. **(B)** Kymographs generated from live-cell imaging assays demonstrating typical neuritic mitochondria movement for vector, LRRK2-WT, and LRRK2-R1441C conditions. **(C)** Quantification of percentage of mitochondria traveling less than 2 microns (stationary), greater than two microns away from cell body (anterograde), or greater than two microns toward cell body (retrograde) in cells expressing GFP for each condition. ^*^p < 0.05 by ANOVA compared to vector control. {*F*_(2, 5)_ = 15.49; *F*_(2, 5)_ = 10.32; *F*_(2, 5)_ = 22.65 for stationary, anterograde, retrograde, respectively}. **(D)** Quantification of total, anterograde, and retrograde run lengths of mitochondria traveling at least 2 microns in GFP-expressing cells for each condition. ^*^p < 0.05 by ANOVA compared to vector control. {*F*_(2, 66)_ = 3.354; *F*_(2, 21)_ = 0.1804; *F*_(2, 42)_ = 3.271 for total, anterograde, retrograde run length, respectively}.

To assess whether mutant LRRK2 alters neural transport, human SH-SY5Y cells were employed to track mitochondrial transport using a live-cell imaging assay. Mitochondria were labeled with MitoTracker Orange and defined as stationary (<2 micron displacement), anterograde (≥2 micron displacement away from cell body), or retrograde (≥2 micron displacement toward cell body) (Figure 2A). Kymographs generated from time-lapse image sequences show a greater tendency for mitochondria in LRRK2-R1441C expressing cells to remain stationary as compared with vector controls or cells expressing wild type-LRRK2 (Figure 2B). LRRK2-R1441C also significantly decreases the number of mitochondria traveling in both anterograde and retrograde directions compared with vector controls (Figure 2C). Cells expressing wild type-LRRK2 did not change compared with control cells. In order to assess the processive nature of mitochondria in the above conditions, run lengths of mitochondria traveling at least 2 microns were measured. Cells expressing LRRK2-R1441C showed a significant decrease in total run length of motile mitochondria compared with cells expressing vector or wild type-LRRK2, with a significant decrease in the retrograde transport (Figure 2D). Wild-type LRRK2 did not affect observed mitochondrial transport in SH-SY5Y cells under live-cell imaging (Figures 2C,D), possibly due to cell auto-modulation to balance the normal transport.

### 68 and FX2149, GTP-binding inhibitors, attenuated LRRK2-R1441C-induced mitochondrial transport defects

**Figure 3:**
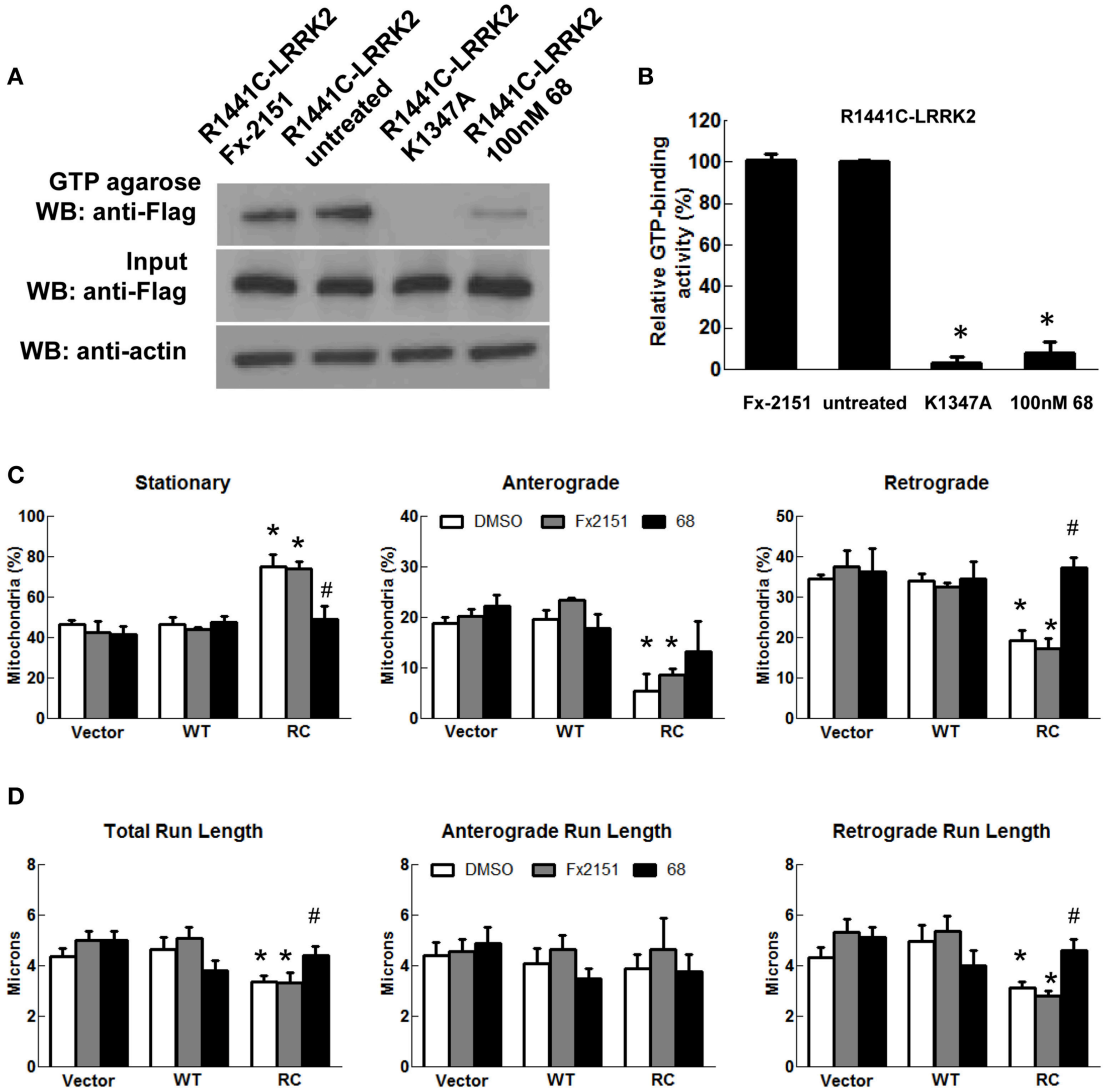
**A GTP-binding inhibitor, 68, improved mitochondria transport impairment in LRRK2-R1441C cells**. SH-SY5Y cells co-transfected with GFP and either Flag-LRRK2-R1441C, Flag-LRRK2-WT, or vector plasmids at a 1:15 ratio were treated with 68 at 100 nM at 4 h post-transfection for 48 h. **(A,B)**. GTP-binding assay of cell lysates. **(A)** Western blots of GTP-binding assays. R1441C/K1347A double mutant represents a non-GTP-binding control lysate. FX2151 represents a 68 analog with no effect on GTP-binding activity (inactive control). **(B)** Quantification of GTP-binding activity from three repeated GTP binding assays. ^*^p < 0.05 by ANOVA compared to R1441C with vehicle treatment. **(C)** Mitochondria events were quantified as stationary, anterograde, or retrograde and expressed as a percentage of total mitochondria for various groups as indicated., ^*^p < 0.05 by ANOVA compared to vector control. {*F*_(2, 5)_ = 15.49; *F*_(2, 5)_ = 10.32; *F*_(2, 5)_ = 22.65 for stationary, anterograde, retrograde, respectively}. ^#^*p* < 0.05 by ANOVA compared to R1441C with vehicle treatment. {*F*_(2, 6)_ = 7.243; *F*_(2, 6)_ = 0.9794; *F*_(2, 6)_ = 20.42 for stationary, anterograde, retrograde, respectively}. **(D)** Quantification of total, anterograde, and retrograde run lengths of mitochondria traveling at least 2 microns in 68 treated, FX2151 treated, and DMSO treated GFP-expressing cells. ^*^p < 0.05 by ANOVA compared to vector control. {*F*_(2, 66)_ = 3.354; *F*_(2, 21)_ = 0.1804; *F*_(2, 42)_ = 3.271 for total, anterograde, retrograde run length, respectively}. ^#^*p* < 0.05 by ANOVA compared to R1441C with vehicle treatment. {*F*_(2, 47)_ = 3.468; *F*_(2, 10)_ = 0.3281; *F*_(2, 34)_ = 6.779 for total, anterograde, retrograde run length, respectively}.

**Figure 4:**
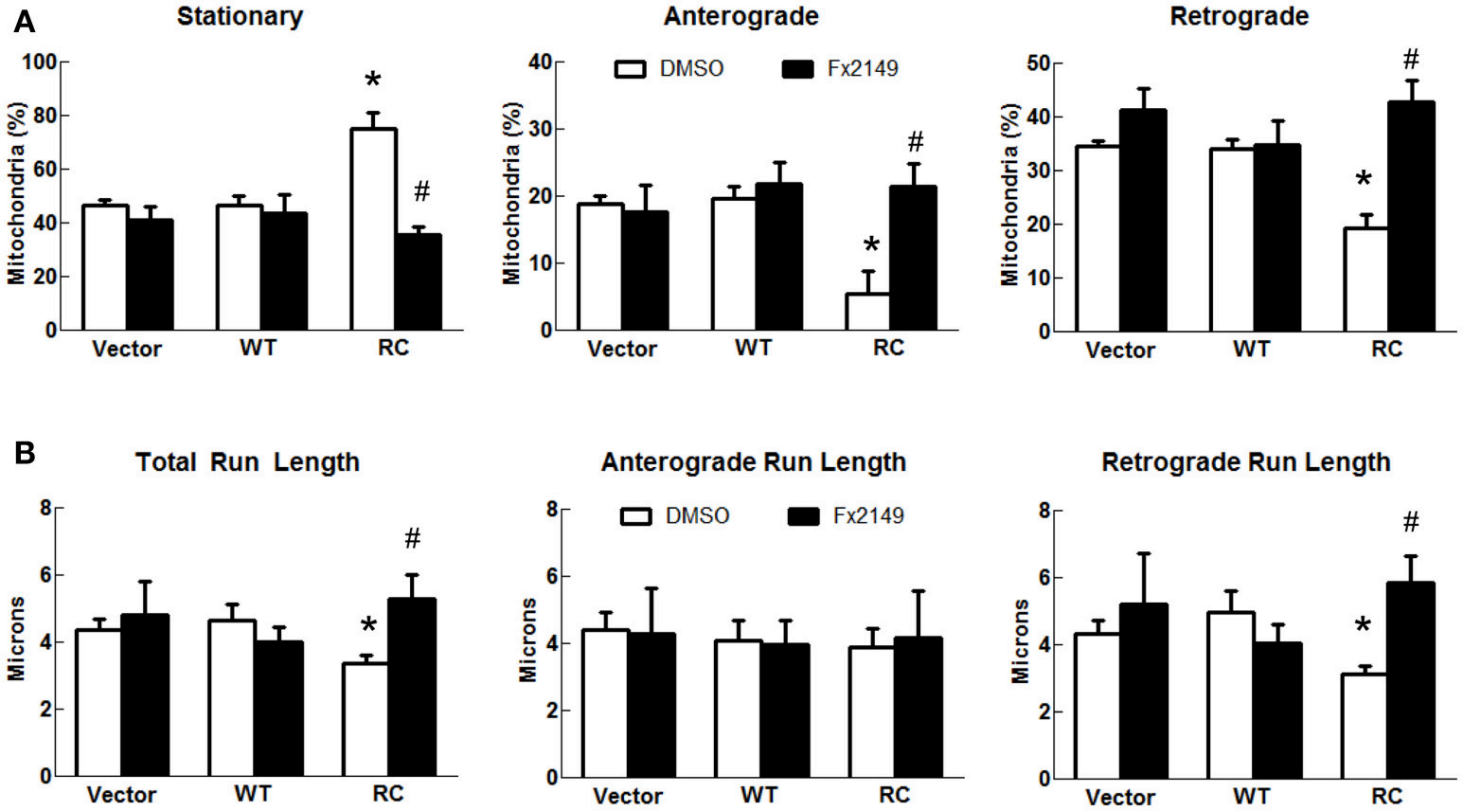
**A GTP-binding inhibitor FX2149 improved mitochondrial transport impairment in LRRK2-R1441C cells**. SH-SY5Y cells co-transfected with GFP and either Flag-LRRK2-R1441C, Flag-LRRK2-WT, or vector plasmids at a 1:15 ratio were treated with FX2149 at 100 nM at 4 h post-transfection for 48 h. **(A)** Mitochondria events were quantified as stationary, anterograde, or retrograde and expressed as a percentage of total mitochondria for DMSO and FX2149 treatments. ^*^p < 0.05 by ANOVA compared to vector control. {*F*_(2, 5)_ = 15.49; *F*_(2, 5)_ = 10.32; *F*_(2, 5)_ = 22.64 for stationary, anterograde, retrograde, respectively}. #*p* < 0.05 by *t*-test compared to R1441C with vehicle treatment. {*F*_(4)_ = 3.699; *F*_(4)_ = 1.049; *F*_(4)_ = 2.855 for stationary, anterograde, retrograde, respectively}. **(B)** Quantification of total, anterograde, and retrograde run lengths of mitochondria traveling at least 2 microns in FX2149 treated and DMSO treated GFP-expressing cells. ^*^p < 0.05 by ANOVA compared to vector control. {*F*_(2, 66)_ = 3.774; *F*_(2, 21)_ = 0.1804; *F*_(2, 42)_ = 3.271 for stationary, anterograde, retrograde run length, respectively}. #*p* < 0.05 by *t*-test compared to R1441C with vehicle treatment. {*F*_(24)_ = 4.974; *F*_(6)_ = 3.828; *F*_(16)_ = 6.340 for stationary, anterograde, retrograde run length, respectively}.

As LRRK2-R1441C mutations occur within its GTPase domain, a novel LRRK2-specific GTP-binding inhibitor recently discovered by our lab (Li et al., 2014, 2015) was used to assess whether it alters mitochondria transport in cells expressing LRRK2-R1441C. Consistent with previous studies (Li et al., 2014), a GTP-binding inhibitor, compound 68, significantly reduces LRRK2-R1441C binding with GTP, while its structural analog, a negative control compound, FX2151, did not (Figures 3A,B). Treatment with 68 did not alter the mitochondrial transport in vector control or LRRK2-WT cells (Figures 3C,D). Interestingly, 68 significantly reduced the number of stationary mitochondria in cells expressing LRRK2-R1441C. Moreover, 68 also improved LRRK2-R1441C-induced impairments of anterograde and retrograde mitochondrial movement in neurites (Figure 3C). 68 had more of an effect on improving mitochondrial transport in the retrograde direction, while only displaying a slight trend in increasing anterograde movement but with no significance (Figures 3C,D). Following treatment with another GTP-binding inhibitor, FX2149, the number of stationary mitochondria was likewise decreased in the RC condition in favor of increased anterograde and retrograde levels (Figure 4). The run lengths were also increased in the RC level toward vector controls following FX2149 treatment, significantly in the retrograde direction. In contrast, there was no effect with negative control compound FX2151 on mitochondrial transport.

### LRRK2-R1441C impaired lysosomal transport in SH-SY5Y neurites is improved by 68 and FX2149

**Figure 5:**
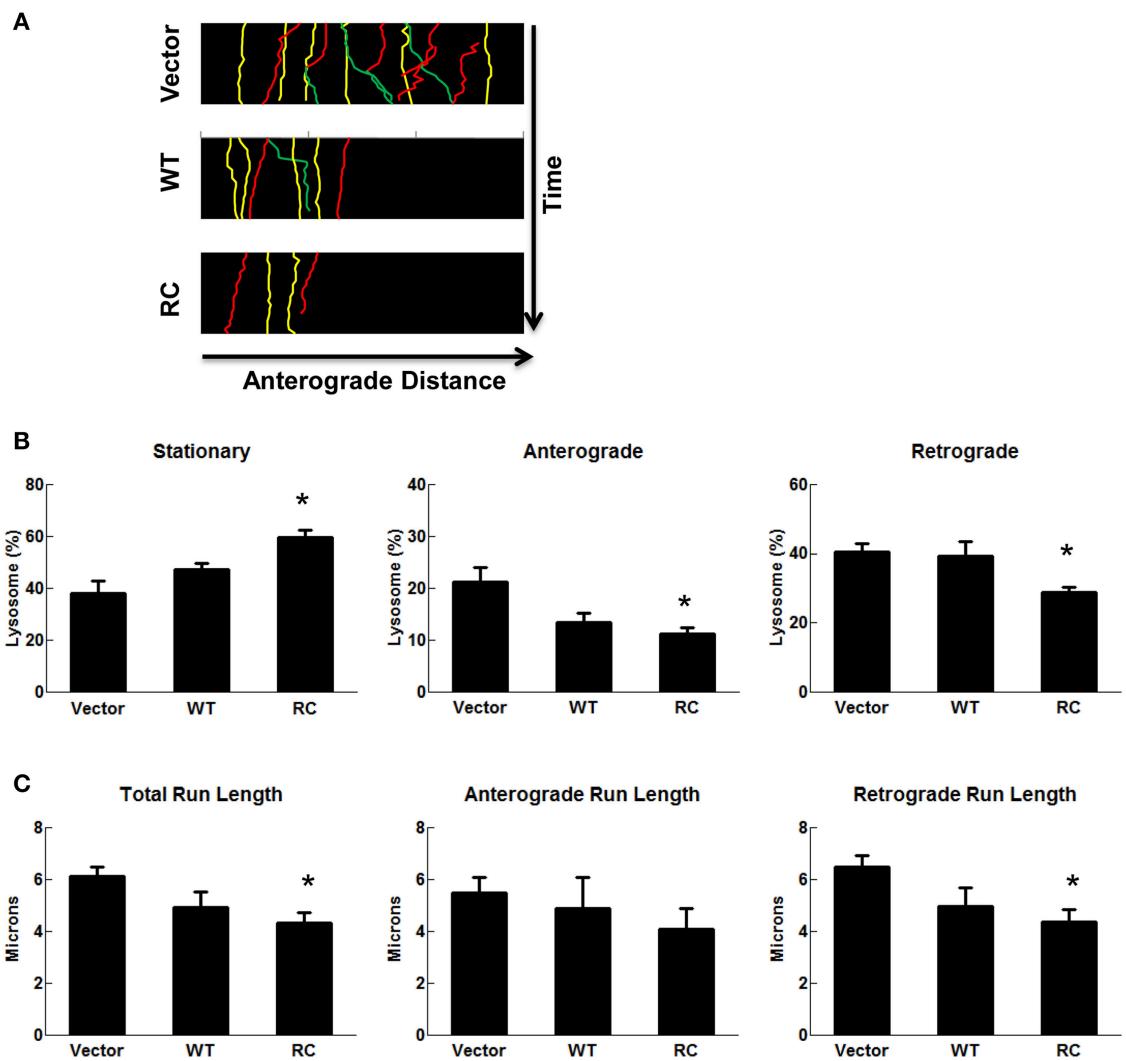
**LRRK2-R1441C induced lysosome transport impairment**. SH-SY5Y cells were co-transfected with GFP and various pcDNA3.1-LRRK2 plasmids at a 1:15 ratio for 48 h. Prior to imaging, cells were stained with LysoTracker Red for 30 min. **(A)** A representative image depicts time-lapse live-cell imaging technique used for measuring neuritic lysosomal transport. Lysosomes were traced using images captured during time progression, and classified as stationary (yellow), anterograde (green), or retrograde (red). Kymographs generated from live-cell imaging assays demonstrating neuritic lysosome movement. Lysosomal events were classified as stationary (yellow), anterograde (green), or retrograde (red). **(B)** Quantification of percentage of lysosomes traveling less than 2 microns (stationary), greater than two microns away from cell body (anterograde), or greater than two microns toward cell body (retrograde) in cells expressing GFP for each condition. ^*^p < 0.05 by ANOVA compared to vector control. {*F*_(2, 7)_ = 8.184; *F*_(2, 7)_ = 5.423, *F*_(2, 7)_ = 5.275 for stationary, anterograde, retrograde, respectively}. **(C)** Quantification of total, anterograde, and retrograde run lengths of lysosomes traveling at least 2 microns in GFP-expressing cells for each condition. ^*^p < 0.05 by ANOVA compared to vector control. {*F*_(2, 103)_ = 4.249; *F*_(2, 32)_ = 0.4790; *F*_(2, 68)_ = 4.707 for total, anterograde, retrograde run length, respectively}.

Lysosome transport patterns were observed using a live-cell imaging approach and characterized as either stationary, anterograde, or retrograde as described above for mitochondrial transport analysis. Expression of wild type-LRRK2 did not alter lysosome transport compared with vector control cells (Figure 5). In contrast, expression of LRRK2-R1441C mutant significantly increased the number of stationary lysosomes and decreased the number of lysosomes in both anterograde and retrograde directions (Figures 5A–C). These results were further validated by the run-length assays of lysosome transport (Figure 5D). A significant decrease in total run lengths of lysosomes traveling at least 2 microns was seen in cells transfected with LRRK2-R1441C (Figure 5D). LRRK2-R1441C caused more reductions in the retrograde direction, while there was only a slight trend in reduction with no significance in anterograde movement in the run-length assays.

**Figure 6:**
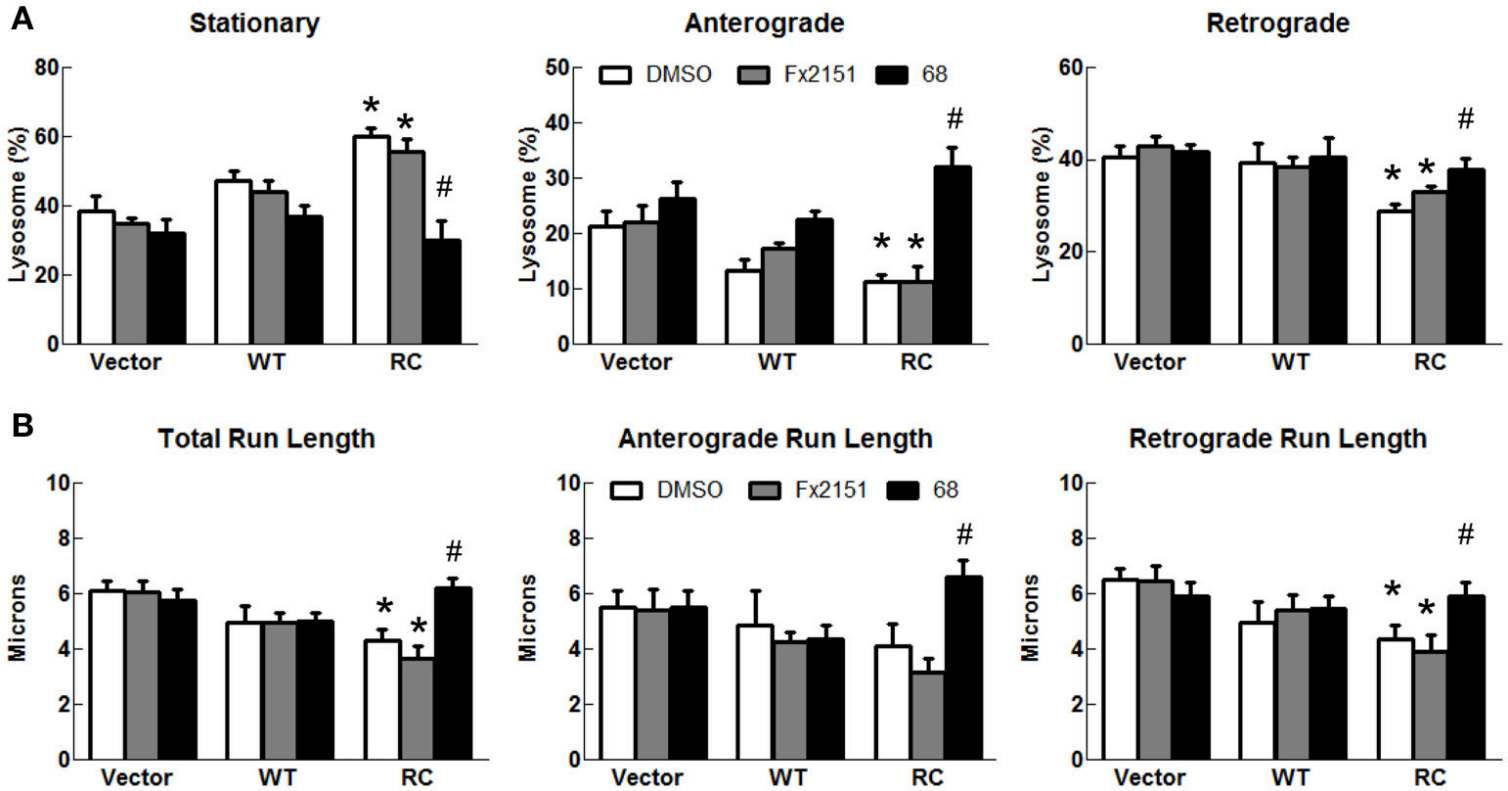
**68 improved LRRK2-R1441C-induced lysosome transport defects**. SH-SY5Y cells co-transfected with GFP and either Flag-LRRK2-R1441C, Flag-LRRK2-WT, or vector plasmids at a 1:15 ratio were treated with 68 at 100 nM at 4 h post-transfection for 48 h. **(A)** Lysosomal events were quantified as stationary, anterograde, or retrograde and expressed as a percentage of total lysosomes for DMSO, FX2151 (100 nM), and 68 treatments (100 nM). ^*^p < 0.05 by ANOVA compared to vector control. {*F*_(2, 7)_ = 8.184; *F*_(2, 7)_ = 5.423, *F*_(2, 7)_ = 5.275 for stationary, anterograde, retrograde, respectively}. ^#^*p* < 0.05 by ANOVA compared to R1441C with vehicle treatment. {*F*_(2, 6)_ = 15.66; *F*_(2, 6)_ = 19.57, *F*_(2, 6)_ = 6.392 for stationary, anterograde, retrograde, respectively}. **(B)** Quantification of total, anterograde, and retrograde run lengths of lysosomes traveling at least 2 microns in 68 treated, FX2151 treated, and DMSO treated GFP-expressing cells. ^*^p < 0.05 by ANOVA compared to vector control. {*F*_(2, 103)_ = 4.249; *F*_(2, 32)_ = 0.4790; *F*_(2, 68)_ = 4.707 for total, anterograde, retrograde run length, respectively}. ^#^*p* < 0.05 by ANOVA compared to R1441C with vehicle treatment. {*F*_(2, 70)_ = 11.91; *F*_(2, 20)_ = 8.789; *F*_(2, 47)_ = 4.666 for total, anterograde, retrograde run length, respectively}.

**Figure 7:**
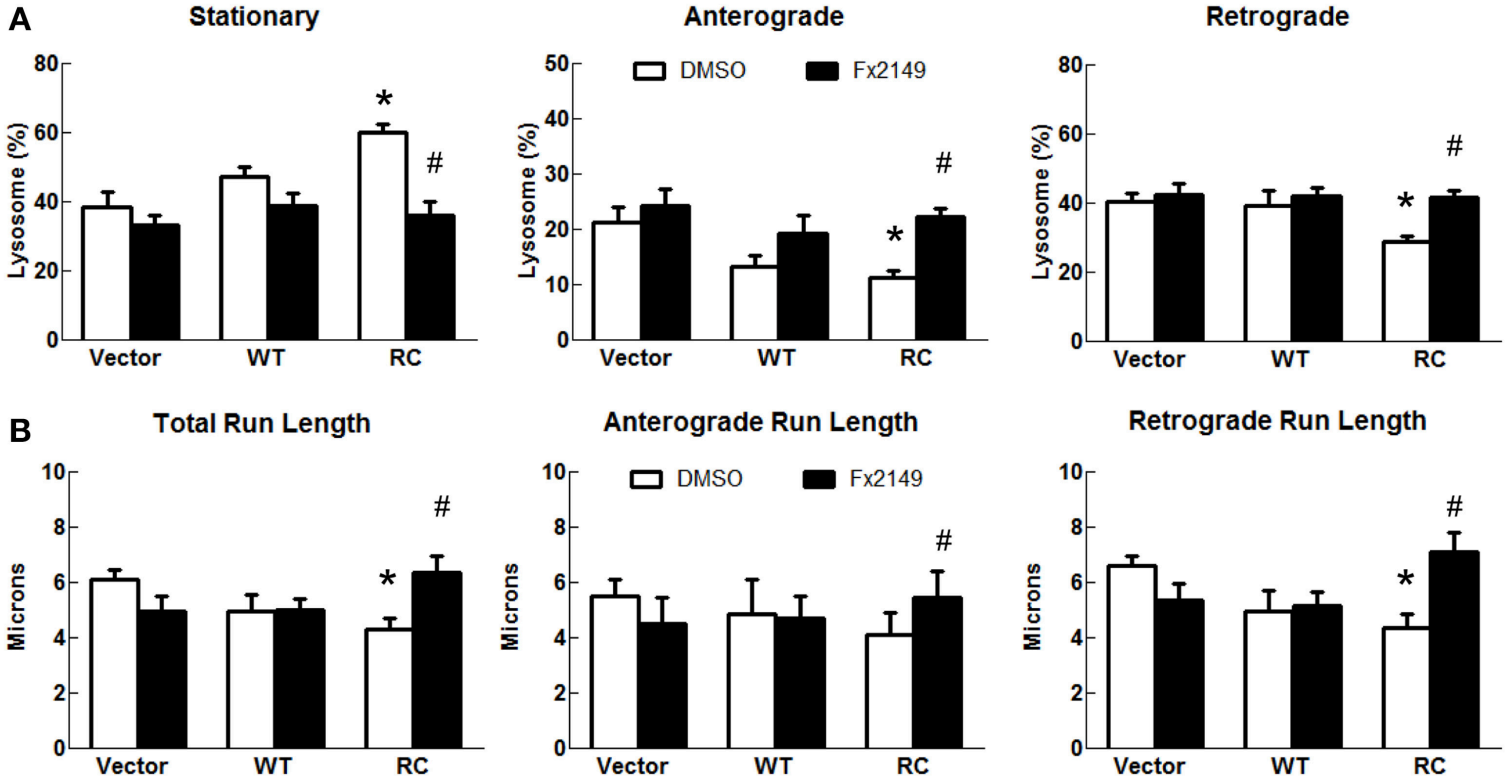
**FX2149 restored lysosomal transport in LRRK2-R1441C cells**. SH-SY5Y cells co-transfected with GFP and either Flag-LRRK2-R1441C, Flag-LRRK2-WT, or vector plasmids at a 1:15 ratio were treated with FX2149 at 100 nM at 4 h post-transfection for 48 h. **(A)** Lysosomal events were quantified as stationary, anterograde, or retrograde and expressed as a percentage of total lysosomes for DMSO and FX2149 treatments. ^*^p < 0.05 by ANOVA compared to vector control. {*F*_(2, 7)_ = 8.18; *F*_(2, 7)_ = 5.426; *F*_(2, 7)_ = 5.510 for stationary, anterograde, retrograde, respectively}. #*p* < 0.05 by *t*-test compared to R1441C with vehicle treatment. {*F*_(4)_ = 2.18; *F*_(4)_ = 1.399; *F*_(4)_ = 1.778 for stationary, anterograde, retrograde, respectively}. **(B)** Quantification of total, anterograde, and retrograde run lengths of lysosomes traveling at least 2 microns in FX2149 treated and DMSO treated cells. ^*^p < 0.05 by ANOVA compared to vector control. {*F*_(2, 103)_ = 4.249; *F*_(2, 32)_ = 0.4790; *F*_(2, 79)_ = 5.747 for stationary, anterograde, retrograde run length, respectively}. #*p* < 0.05 by *t*-test compared to R1441C with vehicle treatment. {*F*_(36)_ = 2.003; *F*_(10)_ = 2.705; *F*_(24)_ = 1.500 for stationary, anterograde, retrograde run length, respectively}.

To assess the effect of 68 on LRRK2-linked lysosome transport, 68 was used to treat cells expressing vector, LRRK2-WT, and LRRK2-R1441C for 48 h. Treatment of 68 did not alter lysosome transport in vector control or LRRK2-WT cells (Figure 6). However, 68 significantly decreased stationary lysosomes in cells expressing LRRK2-R1441C compared with vehicle treated control cells (Figure 6A). Moreover, 68 significantly improved the R1441C-LRRK2-induced defects in lysosome transport in both anterograde and retrograde directions, compared with R1441C expressing cells treated with vehicle alone (Figure 6). Treatment with FX2149 resulted in a decrease in stationary lysosomes, like in the 68-treated condition (Figure 7). A corresponding increase in anterograde and retrograde lysosomes was observed. Lysosome run lengths were significantly increased amongst anterograde and retrograde organelles in the FX2149-treated condition compared to vehicle treated RC cells. There was no effect on lysosome transport following treatment with negative control compound FX2151.

## Discussion

The major findings of this study are that PD-linked GTPase domain mutation LRRK2-R1441C causes impairments in the neuritic transport of both mitochondria and lysosomes in SH-SY5Y cells and that inhibition of GTP-binding with recently identified pharmacological inhibitors, 68 and FX2149, improves these transport defects. To our knowledge, this is the first report that GTP-binding activity is involved in mitochondrial and lysosomal cargo transport in neuritic processes. Our findings provide novel insight into LRRK2-linked neuron degeneration and a potential strategy for PD intervention through inhibition of GTP-binding activity.

Neuritic injury has been shown to result from impaired functions within neurons, including disruption of mitochondrial energy homeostasis, protein clearance pathways, and intracellular transport processes (Chevalier-Larsen and Holzbaur, 2006; Sheng and Cai, 2012; Schwarz, 2013; Amaya et al., 2015). Axon injury has been reported as one of the early neuronal degeneration events occurring way before cell body degeneration in a number of neurodegenerative diseases, including PD (Chevalier-Larsen and Holzbaur, 2006; Maday et al., 2014). Neural transport, especially axonal transport, is necessary for neuronal functions including mitochondrial energy balance, autophagic-lysosomal degradation, and transportation of vesicles and other cargos.

Under physiological conditions, about two-thirds of axonal mitochondria are in a stationary phase, whereas the remaining third are split equally among mitochondria traveling in anterograde or retrograde directions (Sheng and Cai, 2012; Schwarz, 2013; Maday et al., 2014). About half of axonal lysosomes experience diffusive, random back-and-forth motion of insignificant distances, whereas the other half undergo directed motion equally in either anterograde or retrograde directions (Bandyopadhyay et al., 2014; Maday et al., 2014). Our results demonstrated that expression of the PD-linked mutant, LRRK2-R1441C, significantly increases the number of stationary mitochondria in the neurties of SH-SY5Y cells. Moreover, LRRK2-R1441C reduced the number and distance of mitochondria being transported in both anterograde and retrograde directions. Interestingly, previous reports showed that the LRRK2-G2019S kinase domain mutant does not affect mitochondrial transport in cultured neurons (Godena et al., 2014), suggesting that kinase domain function may not be involved in neural transport. A number of reports showed that disease-linked LRRK2 mutants cause mitochondrial dysfunctions (Cherra et al., 2013; Su and Qi, 2013; Saez-Atienzar et al., 2014; Su et al., 2015), however the underlying mechanisms are unclear. Our results suggest that impairment of the neuritic transport of mitochondria could be one of the early events and mechanisms for mutant LRRK2-inducing mitochondrial dysfunctions and thereby inducing neuritic injury, although this awaits further investigation.

Mutant LRRK2 has been shown to cause lysosome/autophagic pathway impairment (Alegre-Abarrategui and Wade-Martins, 2009; Henry et al., 2015; Esteves and Cardoso, 2016). A recent study has identified that LRRK2 mutations lead to an increase in lysosomal size and a decrease in their ability for degradation (Bandyopadhyay et al., 2014), as well as impairs the autophagic cycle's ability to clear toxic proteins (Alegre-Abarrategui and Wade-Martins, 2009; Esteves and Cardoso, 2016). A functioning axonal transport system is necessary for the lysosome/autophagic pathway, as coordinated lysosome and autophagosome positioning and recruitment are prerequisites (Amaya et al., 2015). Our findings showed that expression of mutant LRRK2 impairs lysosome transport especially in the retrograde direction. These results demonstrate that mutant LRRK2 disruption of lysosomal transport is one of the early events in neuritic injury. LRRK2 has been implicated as a crossroad for the autophagic clearance of mitochondria (mitophagy) (Saez-Atienzar et al., 2014; Esteves and Cardoso, 2016). PD-linked LRRK2 mutations can cause a loss of mitochondrial membrane potential, which precedes elimination by mitophagy, thereby resulting in a decrease in available mitochondria for energy requirements (Su et al., 2015). These mutations also dysregulate fission and fusion events, leading to an increase in mitochondria fragmentation which can trigger an excessive need for autophagy (Wang et al., 2012; Su and Qi, 2013). Thus, mutant LRRK2 impairs both mitochondrial and lysosomal transport, which could trigger lysosome/autophagy pathway impairment resulting in eventual degeneration.

The GTPase domain of LRRK2 has been previously identified as a druggable entity, with GTP-binding inhibitors as potential pharmaceutical candidates (Li et al., 2014, 2015). Pathological LRRK2 mutations have been shown to lead to altered enzymatic activities, and we recently have identified novel GTP-binding inhibitors, 68 and FX2149, which reduces LRRK2 kinase activity and protects against mutant-LRRK2-induced neurodegeneration (Li et al., 2014; Raquel Esteves et al., 2014). In this study, we found that 68 and FX2149 can significantly attenuate mutant LRRK2-induced impairment of mitochondrial and lysosomal transport in SH-SY5Y neurites. These findings suggest that LRRK2 GTP-binding activity is critical for neuritic transport. The machinery of intracellular organelle transport and how neurons specifically regulate transport processes is a complex system (Chevalier-Larsen and Holzbaur, 2006; Fu and Holzbaur, 2014; Maday et al., 2014; Wong and Holzbaur, 2014). Kinesins and dynein motor proteins participate in active anterograde and retrograde transport, respectively, by “stepping” along microtubules (Maday et al., 2014). This is done through an intricate process of acetylation and deacetylation of the tubulin components of the cytoskeleton, which the GTPase domain of LRRK2 has been shown to affect (Godena et al., 2014; Law et al., 2014). LRRK2-R1441C is in the GTPase domain and is implicated in disrupting the balance of autophagic structures, which rely on functioning transport systems (Alegre-Abarrategui et al., 2009; Orenstein et al., 2013). Our studies with GTP-binding inhibitors, 68 and FX2149, show an almost completely rescued lysosomal transport impairment in these mutations, suggesting that GTPase domain activity plays a critical role in mutant LRRK2-R1441C-induced transport defects. LRRK2 GTP-binding activity has been reported as critical for LRRK2 action, and inhibition of GTP-binding could ameliorate neurotoxicity resulting from R1441C-induced dysfunction (Lewis et al., 2007; Xiong et al., 2010; Taymans, 2012; Tsika and Moore, 2013). Pharmacological inhibition of GTP-binding in LRRK2 could improve neural transport functions and protect neurons against neurite injury and degeneration. These findings further indicate that inhibition of GTP-binding activity could be a valuable therapeutic approach for PD intervention.

In conclusion, our work demonstrates consequential mitochondria and lysosome neuritic transport impairments as a result of LRRK2-R1441C mutations, with a corresponding neurite injury phenotype in SH-SY5Y cells. Inhibition of LRRK2 GTP-binding activity by compounds 68 and FX2149 improved mutant LRRK2-R1441C-induced transport defects. As such, targeting the GTP-binding region of LRRK2 may be a potential therapy for LRRK2-related PD onset.

## Author contributions

JT designed the research, collected primary data, performed analysis, and wrote the manuscript. TL was responsible for primary data collection and analysis. WY and FX were responsible for primary data collection and synthesis of FX2149 compound. PF and WS were responsible for designing the research and data analysis. WS is the corresponding author for this study.

### Conflict of interest statement

The authors declare that the research was conducted in the absence of any commercial or financial relationships that could be construed as a potential conflict of interest. The pharmacological uses of 68 and FX2149 are included in a patent by the University of Maryland.
